# *In vivo* dynamic analysis of BMP-2-induced ectopic bone formation

**DOI:** 10.1038/s41598-020-61825-2

**Published:** 2020-03-16

**Authors:** Kunihiko Hashimoto, Takashi Kaito, Masayuki Furuya, Shigeto Seno, Daisuke Okuzaki, Junichi Kikuta, Hiroyuki Tsukazaki, Hideo Matsuda, Hideki Yoshikawa, Masaru Ishii

**Affiliations:** 1 Department of Immunology and Cell Biology, Graduate School of Medicine & Frontier Biosciences, Osaka University, Osaka, 565-0871 Japan; 2 Department of Orthopaedic Surgery, Graduate School of Medicine, Osaka University, Osaka, 565-0871 Japan; 3 Department of Orthopaedic Surgery, Japan Organization of Occupational Health and Safety Osaka Rosai Hospital, Osaka, 591-8025 Japan; 4 Department of Bioinformatic Engineering, Graduate School of Information Science & Technology, Osaka University, Osaka, 565-0871 Japan; 5 Genome Information Research Center, Research Institute for Microbial Diseases, Osaka University, Osaka, 565-0871 Japan

**Keywords:** Biomaterials, Biomedical engineering, Imaging techniques, Bone

## Abstract

Bone morphogenetic protein (BMP)-2 plays a central role in bone-tissue engineering because of its potent bone-induction ability. However, the process of BMP-induced bone formation *in vivo* remains poorly elucidated. Here, we aimed to establish a method for intravital imaging of the entire process of BMP-2-induced ectopic bone formation. Using multicolor intravital imaging in transgenic mice, we visualized the spatiotemporal process of bone induction, including appearance and motility of osteoblasts and osteoclasts, angiogenesis, collagen-fiber formation, and bone-mineral deposition. Furthermore, we investigated how PTH1-34 affects BMP-2-induced bone formation, which revealed that PTH1-34 administration accelerated differentiation and increased the motility of osteoblasts, whereas it decreased morphological changes in osteoclasts. This is the first report on visualization of the entire process of BMP-2-induced bone formation using intravital imaging techniques, which, we believe, will contribute to our understanding of ectopic bone formation and provide new parameters for evaluating bone-forming activity.

## Introduction

Biological enhancement of bone formation is performed for repairing critical-sized bone defects caused by trauma or tumor resection, as well as for fusion surgery and correction of spinal disorders. Delay or failure in bone fusion may prolong the deterioration of quality of life and requirement for additional surgery, and might even adversely affect life expectancy^1,2^.

Bone morphogenetic proteins (BMPs), which are members of the transforming growth factor-β superfamily, play a central role in bone-tissue engineering, as BMPs can potently induce bone growth^3^; however, the dose-dependent side effects related to inflammatory response has prevented the widespread use of BMPs^4^. Although several treatment approaches have been attempted, including the use of sustained drug-delivery systems^5–7^ and combined use with other growth factors^8–10^, harnessing the bone-induction capacity of BMPs efficiently while concurrently minimizing the side effects remains challenging. One potential reason for the difficulty in optimizing BMP-induced bone formation is the poor understanding of the bone-induction process *in vivo*.

Over the past two decades, intravital two-photon microscopy, which can penetrate thick specimens, has launched a new era in the field of biological imaging^11^. Using this innovative technique, *in vivo* interactions between osteoblasts (OBs), osteoclasts (OCs), and immune cells have been demonstrated in the calvarial bone^12–14^.

The ectopic bone-induction process triggered by bone-tissue engineering follows a non-physiological pathway of bone formation and hence should be evaluated separately from normal skeletogenesis. Here, we aimed to develop a technique for intravital imaging of an entire process of BMP-2-induced ectopic bone formation. We established a method for intravital imaging of the BMP-2-induced ectopic bone formation process, in which OBs were visualized in transgenic mice expressing enhanced cyan fluorescent protein (ECFP) in OBs, and, concomitantly, collagen fibers (CFs), bone mineralization, and blood vessels were also visualized. This imaging method will provide novel insights, which will enhance our understanding regarding the *in vivo* dynamic mechanism of BMP-2-induced bone formation.

## Results

### Intravital imaging of ectopic bone formation

**Figure 1:**
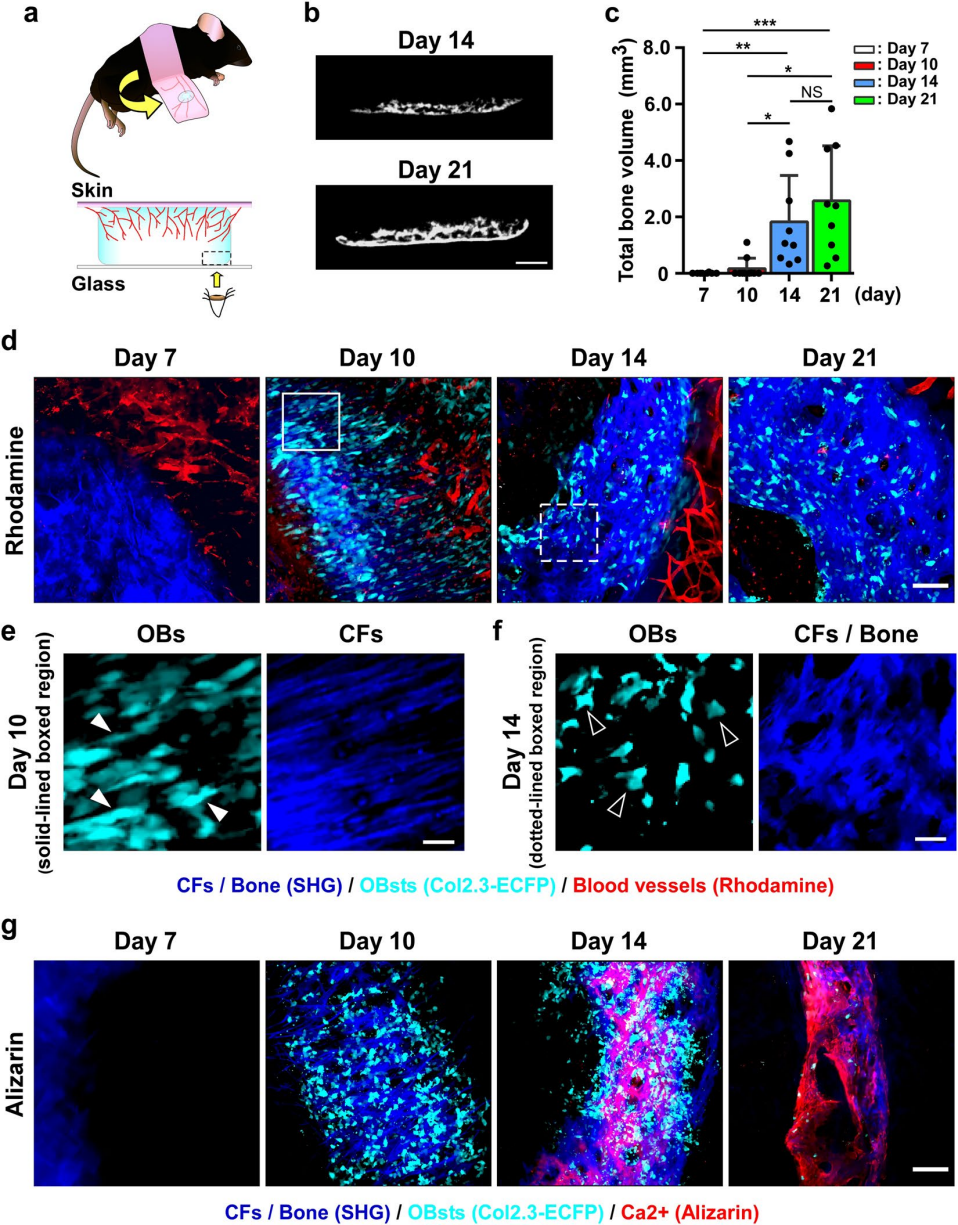
Establishment of intravital two-photon imaging for ectopic bone formation. (**a**) Schematic showing flipped skin with a U-shaped incision at the site of CS implantation (upper panels). Magnified schematic images of ectopic bone formation area (lower panels). Red lines: angiogenesis from surrounding tissue to CS; bone formation (in blue) starts from the CS rim. Area enclosed by dotted line: area examined using two-photon microscopy. The images were created Canvas software (version X 16, https://www.canvasgfx.com/). (**b**,**c**) Micro-CT analysis of ectopic bone 7, 10, 14, and 21 days after CS implantation. (**b**) Representative micro-CT images of ectopic bone on days 14 and 21. Scale bar, 1 mm. (**c**) Total bone volume. n = 8–10/group. (**d**) Representative intravital two-photon microscopy images of implanted CS in *Col2.3*-ECFP mice from day 7 to day 21. Cyan: OBs expressing Col2.3-ECFP; red: blood vessels stained with rhodamine; blue: CFs/bone (SHG). Scale bar, 100 μm. (**e**,**f**) Magnified images from (**d**) showing two types of OB morphology and CF orientation. Scale bar, 25 μm. (**e**) Representative images of spindle-shaped OBs (filled arrowheads) and anisotropic CF orientation [region outlined in (**d**)]. (**f**) Representative images of cuboidal OBs (open arrowheads) and isotropic CF orientation [region delineated by dotted lines in (**d**)]. (**g**) Representative intravital two-photon microscopy images of implanted CS in *Col2.3*-ECFP mice from day 7 to day 21. Cyan: OBs expressing Col2.3-ECFP; red: calcium stained with alizarin; blue: CFs/bone (SHG). Scale bar, 100 μm. Data are presented as means ± SD. *P < 0.05; **P < 0.01; ***P < 0.001; NS, not significant (Kruskal-Wallis test).

Subcutaneous implantation of a BMP-2-containing collagen sponge (CS)^15^, which is an established animal model for BMP-2-induced ectopic bone formation, was used (Fig. 1a, Supplementary Fig. S1a). Induction of ectopic bone formation in this model using 2.5 μg recombinant human (rh) BMP-2 was confirmed based on micro-computed tomography (micro-CT) and histology (Supplementary Fig. S1b,c). CS samples containing 2.5 μg BMP-2 were implanted between the skin and fascia of dorsal muscle in Col1a1*2.3-ECFP mice (*Col2.3*-ECFP mice), and at days 7, 10, 14, and 21 after surgery, micro-CT analysis and intravital imaging were performed (Supplementary Movie 1). Micro-CT results demonstrated that the volume of the induced bone increased with time until day 21 (Fig. 1b,c), and intravital imaging revealed OB recruitment (ECFP; cyan), CF formation (second harmonic generation, SHG; blue), angiogenesis (rhodamine; red) (Fig. 1d–f, Supplementary Movie 2), and CF mineralization (alizarin; red) (Fig. 1g) at each time point. On day 7, when the implanted CS was still detected, blood vessels were formed around the CS. On day 10, spindle-shaped OBs were recruited, and concomitant formation of CFs, which were oriented towards the central part of the CS, was observed (Fig. 1e). The CF orientation became obscure over time, and this could be clearly distinguished from the remnant of the implanted CS. From day 14 onwards, OB morphology also changed and became cuboid in shape (Fig. 1f), and positive alizarin staining—indicating mineral deposition on CFs—was confirmed, with the staining being more prominent on day 21. These results demonstrated that our imaging method can be successfully used to visualize the process of BMP-2-induced ectopic bone formation.

### Dynamic changes in induced OBs during ectopic bone formation

**Figure 2:**
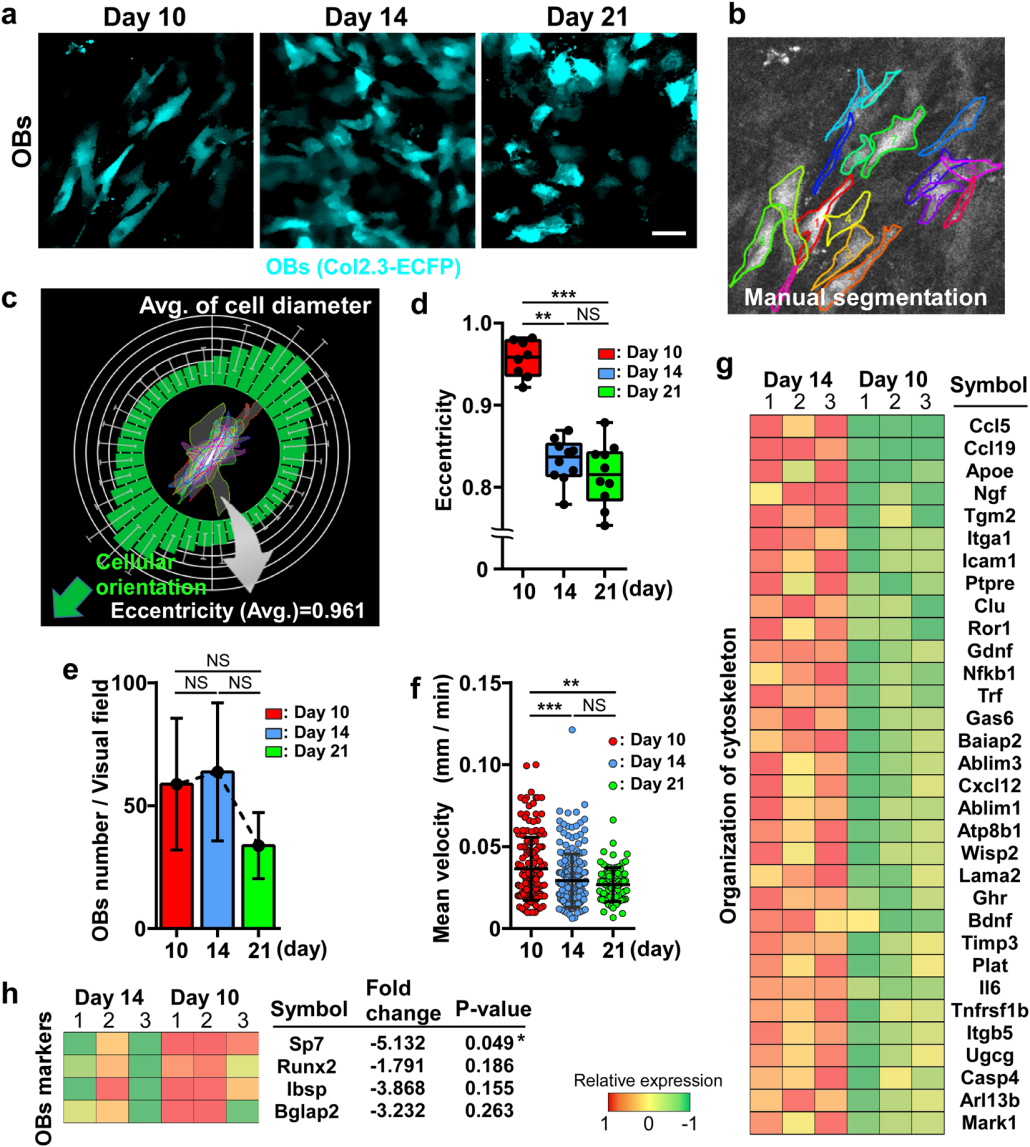
Changes in OB morphology and motility during ectopic bone formation. (**a**) Temporal change in OB morphology in *Col2.3*-ECFP mice from day 10 to day 21. Cyan: OBs expressing *Col2.3*-ECFP. Scale bar, 25 μm. (**b–d**) Image analysis method for quantifying changes in OB morphology based on eccentricity. (**b**) Manually detected OBs [image from day 10 in (**a**)]. (**c**) Example of the average value of eccentricity measured for each cell from day 10 in the image shown in (**a**). The eccentricity value of 0.961 indicated that cellular shape was close to a straight line. The image was created by R (version 3.5.0, https://www.r-project.org/). (**d**) Eccentricity measurement from day 10 to day 21 after CS implantation. n = 8–10, representative of images collected from 4–5 mice/group. (**e**) Changes in the number of OBs/visual field. n = 4/group. (**f**) Mean velocity of OBs/visual field. Data points (n = 168, 208, and 83 on days 10, 14, and 21, respectively) represent single cells collected from three mice/group. Data are presented as means ± SD. **P < 0.01; ***P < 0.001; NS, not significant (Kruskal-Wallis test). (**g**,**h**) Heat map showing expression levels for a subset of genes that were differentially expressed on days 10 and 14 in ectopic bone. The images were created by Canvas software (version X 16, https://www.canvasgfx.com/). (**g**) Genes associated with ‘organization of cytoskeleton’. (**h**) genes associated with ‘osteoblastic differentiation’. n = 3/group. Data are presented as means ± SD. *P < 0.05 (Mann-Whitney test).

During the ectopic bone formation process, OB morphology exhibited dynamic changes (Fig. 1e,f). To quantify these changes (Fig. 2a), we calculated the eccentricity of OBs (range: 0–1) as a measure of cellular shape (Fig. 2b,c); here, a high eccentricity value (close to 1) indicates linear morphology of OBs, whereas a low value indicates circular morphology. OB eccentricity decreased over time until day 21 (Fig. 2d). Furthermore, the number and the motility of OBs in the visual field were measured at 10-min intervals for 3 h, with motility being measured as the mean velocity using a tracking system (Supplementary Fig. S2a). OBs appeared on day 10 and their number peaked on day 14, after which the number showed a decreasing trend until day 21 (Fig. 2e). Conversely, the mean velocity of OBs decreased over time; the velocity on day 10 was markedly higher than those on days 14 and 21 (Fig. 2f). Collectively, these results indicated that the OB shape became increasingly more cuboidal, although the number and motility of OBs decreased with increase in bone volume during BMP-2-induced ectopic bone formation.

### Gene expression associated with morphological changes in OBs

We examined the differences in gene expression in OBs between day 10 and day 14, a period during which active morphological change and mineralization were observed in the BMP-2 group. Fluorescence-activated cell sorting (FACS) was used to isolate ECFP^+^ OBs in the lesions with ectopic bone formation (Supplementary Fig. S2b), and transcriptome profiling using RNA-sequencing (RNA-seq) was performed to identify changes in gene expression in OBs. Out of 23,284 genes, 182 genes showed an adjusted fold-change of > 2.0 and an FPKM (fragments per kilobase of exon per million reads mapped) of > 0.1 from day 10 to day 14 (data not shown). Among the 182 genes, 32 genes associated with ‘organization of cytoskeleton’ defined by the IPA software were upregulated on day 14 (Fig. 2g). These data support the results of intravital imaging, showing that active morphological changes occurred during this period, although cell motility was affected by several factors other than gene expression.

Here, we also confirmed the expression of canonical OB-marker genes: distal-less homeobox 5 (*Dlx5*), runt-related transcription factor 2 (*Runx2*), Sp7 transcription factor (*Sp7*), alkaline phosphatase, liver/bone/kidney (*Alpl*), collagen, type I, alpha 1 (*Col1a1*), integrin-binding sialoprotein (*Ibsp*), and bone gamma-carboxyglutamate protein 2 (*Bglap2*). *Sp7* was downregulated on day 14; however, no change was detected in any other OB marker (Fig. 2h, Supplementary Fig. S2c).

These results suggested that OBs had already differentiated into mature OBs on day 10 and that this was preceded by their morphological change from spindle-shaped to cuboid shape.

### Orientations of CFs and OBs and their correlation

**Figure 3:**
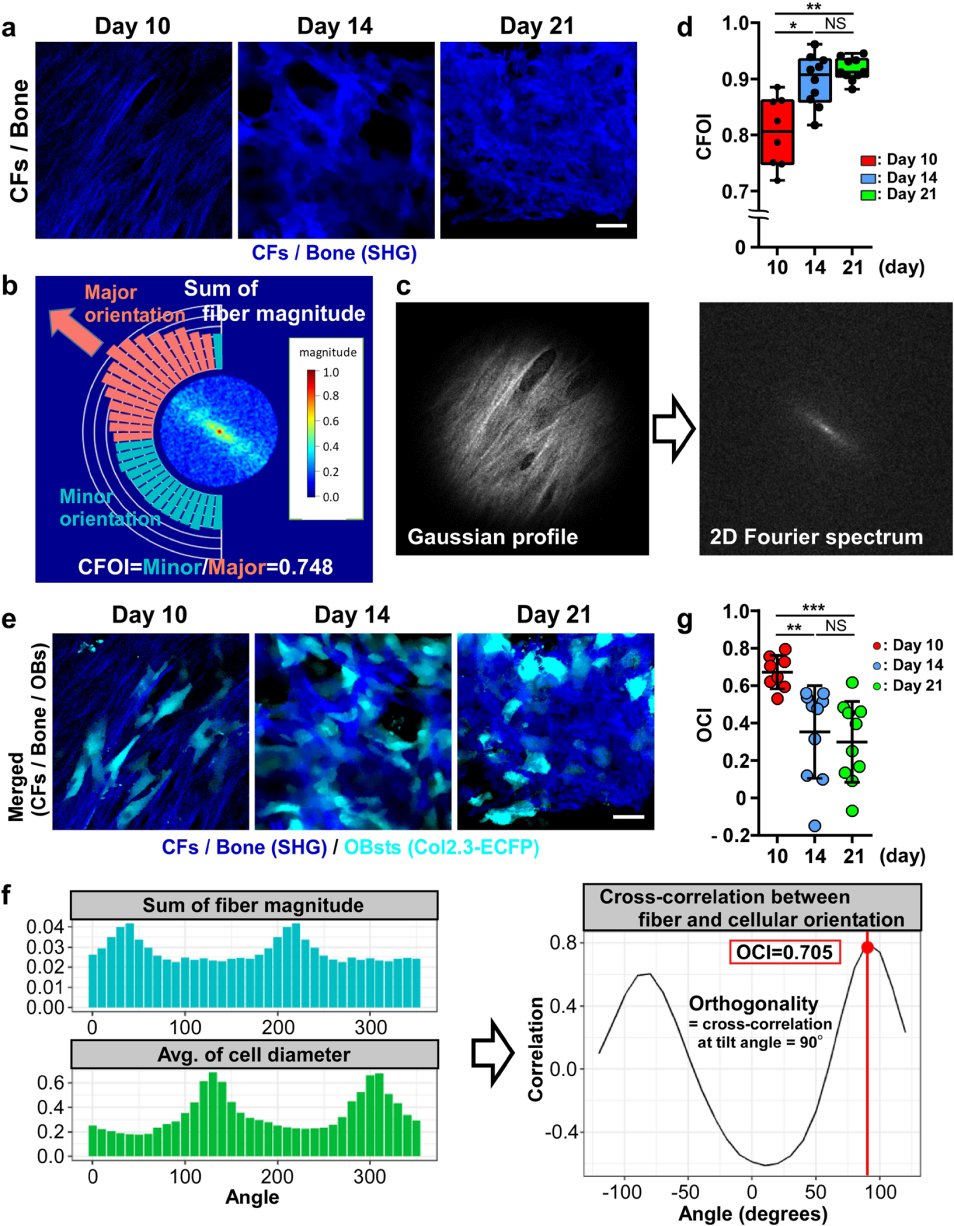
Changes in the orientation of CFs and OBs during ectopic bone formation. (**a**) Temporal change in CF formation in *Col2.3*-ECFP mice from day 10 to day 21. Blue: CFs/bone (SHG). Scale bar, 25 μm. (**b–d**) Image-analysis method for CFOI calculation based on Fourier transformation. (**b**) Example of CFOI calculation for the day 10 image shown with cylindrical Fourier spectrum. The normalized magnitude of the spectrum is shown in a color-coded form. Our method calculates the sum of the magnitude for each angle (shown by the bar plot) and searches the direction of the major orientation; once the major orientation is decided, the sums of the magnitude in the major orientation (red bars) and minor orientation (cyan bars) are obtained and their ratio is calculated. The CFOI value here of 0.748 indicates parallel orientation of the CFs. The image was created by R (version 3.5.0, https://www.r-project.org/). (**c**) Images of only SHG channels [day 10 image in (**a**)] were converted to grayscale (left panel). Power spectrum was obtained using 2D Fourier transformation (right panel). (**d**) CFOI from day 10 to day 21 after CS implantation. n = 8–10, representative of images collected from 4–5 mice/group. (**e**) Temporal change of CF and OB orientations in *Col2.3*-ECFP mice from day 10 to day 21. Blue: CFs/bone (SHG); cyan: OBs expressing *Col2.3*-ECFP. Scale bar, 25 μm. (**f**,**g**) Image analysis method for quantifying CF orientation against OB orientation based on cross-correlation. (**f**) OCI calculated for the image shown in (**b**) and the image shown in (**c**). The OCI value of 0.705 indicates that CF and OB orientations are both anisotropic. (**g**) OCI from day 10 to day 21 after CS implantation. n = 8–10, representative of images collected from 4–5 mice/group. Data are presented as means ± SD. *P < 0.05; **P < 0.01; ***P < 0.001; NS, not significant (Kruskal-Wallis test).

We next focused on the orientational correlation between OBs and the CFs that are produced by OBs (Fig. 1e,f). To quantify the changes in the orientation of newly formed CFs (Fig. 3a), we first calculated a collagen fiber orientation index (CFOI; range: 0–1) based on Fourier transformation (Fig. 3b,c)^16^. High CFOI value (close to 1) indicated that the CFs are randomly oriented (isotropic) in a field of view, whereas low CFOI value indicated that CFs are oriented in the same direction (anisotropic). The calculated CFOI increased over time until day 21, which suggested a time-dependent loss of CF orientation (Fig. 3d). Thus, both the CFOI and the eccentricity indicated marked changes between days 10 and 14.

The orientational correlation between CFs and OBs (Fig. 3e), defined as an orientation correlated index (OCI, ranging from -1 to 1), was investigated using a cross-correlation method (Fig. 3f); high OCI value (close to 1) indicated strong correlation between the degrees of orientation of CFs and OBs. The OCI was found to decrease over time until day 21 (Fig. 3g).

These results indicated that the anisotropic orientation of CFs and the high eccentricity (spindle-shape) of OBs are coordinated during the early stage of ectopic bone formation.

### Effects of PTH administration on ectopic bone formation

**Figure 4:**
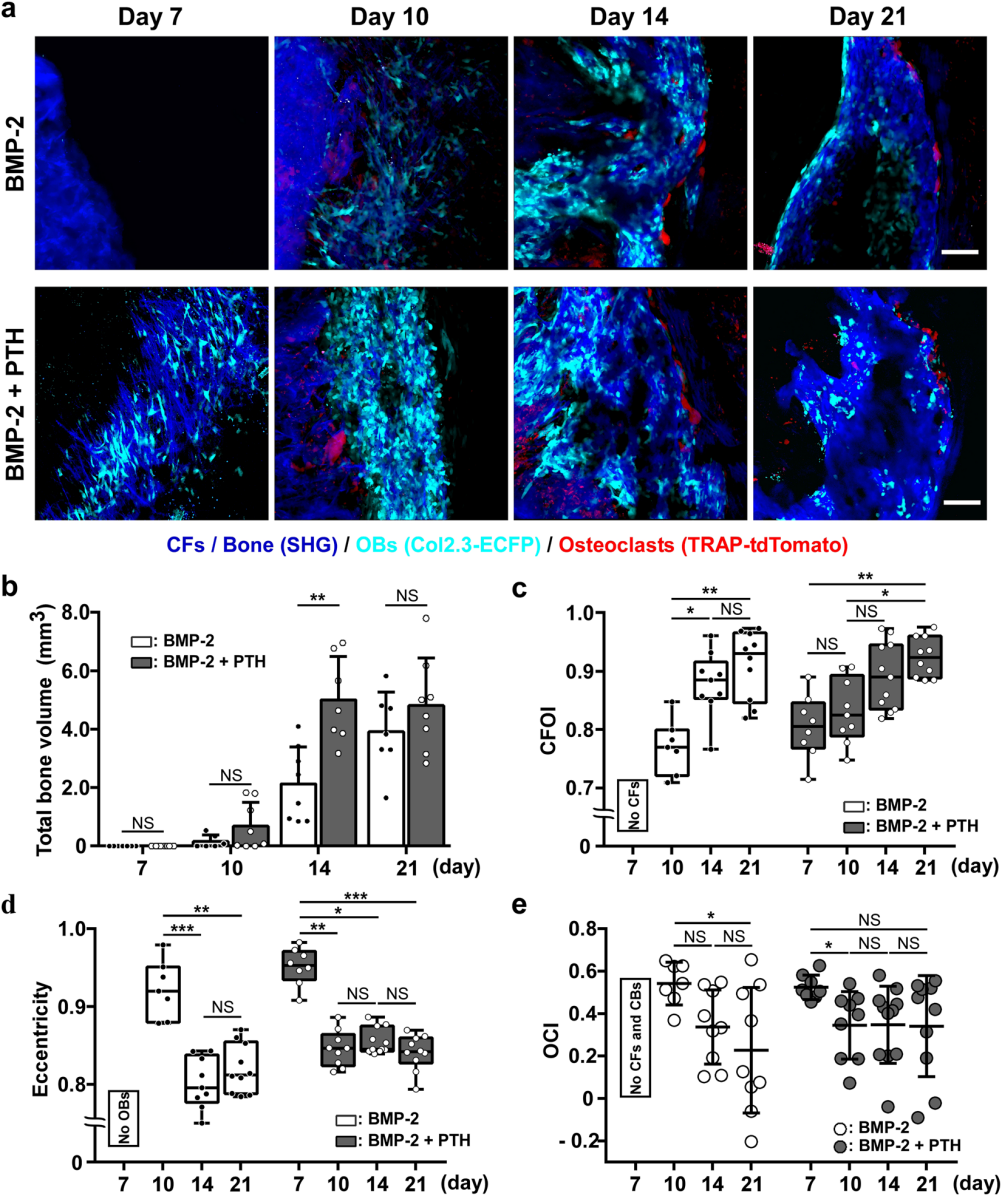
Accelerated ectopic bone formation induced by intermittent administration of PTH. (**a**) Representative intravital two-photon microscopy images of ectopic bone formation in untreated (upper panels) and PTH-treated (lower panels) *Col2.3*-ECFP/TRAP-tdTomato mice from day 7 to day 21 after CS implantation. Cyan: OBs expressing *Col2.3*-ECFP; red: OCs expressing TRAP-tdTomato; blue: CFs/bone (SHG). Scale bar, 100 μm. (**b**) Micro-CT analysis of ectopic-bone volume, with and without PTH treatment, at 7, 10, 14, and 21 days after CS implantation. n = 6–8/group. (**c**) Time-dependent changes in CFOI, with and without PTH administration. n = 7–11, representative of images collected from 4–5 mice/group. (**d**) Eccentricity from day 7 to day 21 after CS implantation. n = 7–11, representative of images collected from 4–5 mice/group. (**e**) OCI from day 7 to day 21 after CS implantation. n = 7–11, representative of images collected from 4–5 mice/group. Data are presented as means ± SD. *P < 0.05; **P < 0.01; ***P < 0.001; NS, not significant (Mann-Whitney test).

PTH is the only anabolic agent that has been approved for the treatment of osteoporosis^17,18^. PTH administration accelerates osteoblastic differentiation and suppresses apoptosis of OBs, leading to an increase in bone mass^19,20^. Furthermore, PTH has been reported to exert synergistic effects on BMP-2-induced ectopic bone formation^10,21–23^. To further understand the synergistic action of PTH, we examined the dynamic effects of PTH treatment on BMP-2-induced bone formation. In this analysis, double-fluorescent-reporter bearing *Col2.3*-ECFP/TRAP-tdTomato mice (TRAP: tartrate-resistant acid phosphatase) received CS implantation and were then subjected to intravital imaging on days 7, 10, 14, and 21. PTH administration (40 μg/kg/day, 5 days/week)^13,24,25^ was initiated 1 week before CS implantation and continued until the end of observation. In untreated mice, OBs and the formation of centrally oriented CFs were initially observed on day 10, the same time point as in *Col2.3*-ECFP mice. OCs also appeared on day 10 in and around the induced bone, and the localization of OCs shifted to the outer edge of the induced bone over time. Notably, PTH administration accelerated both OB differentiation and CF formation, which were detected on Day 7; in contrast, PTH did not affect OC differentiation (on day 10) (Fig. 4a).

PTH-induced increase in bone volume was confirmed using micro-CT and histological analyses, which revealed markedly elevated bone volume in PTH-treated mice on day 14 (Fig. 4b, Supplementary Fig. S3a,b).

We also investigated how PTH treatment affected CF orientation and OB morphology (Supplementary Fig. S4). In accordance with the earlier formation of CFs and differentiation of OBs (observed on day 7), in response to PTH administration, the CFOI, eccentricity, and OCI values could be calculated in the PTH group but not in the control group on day 7 (Fig. 4c–e). Furthermore, the subsequent changes in the CFOI and eccentricity values were also accelerated by PTH administration. These results suggested that PTH administration enhanced BMP-2-induced bone formation by stimulating the differentiation of OBs.

### Effects of PTH on number and motility of OBs and OCs

**Figure 5:**
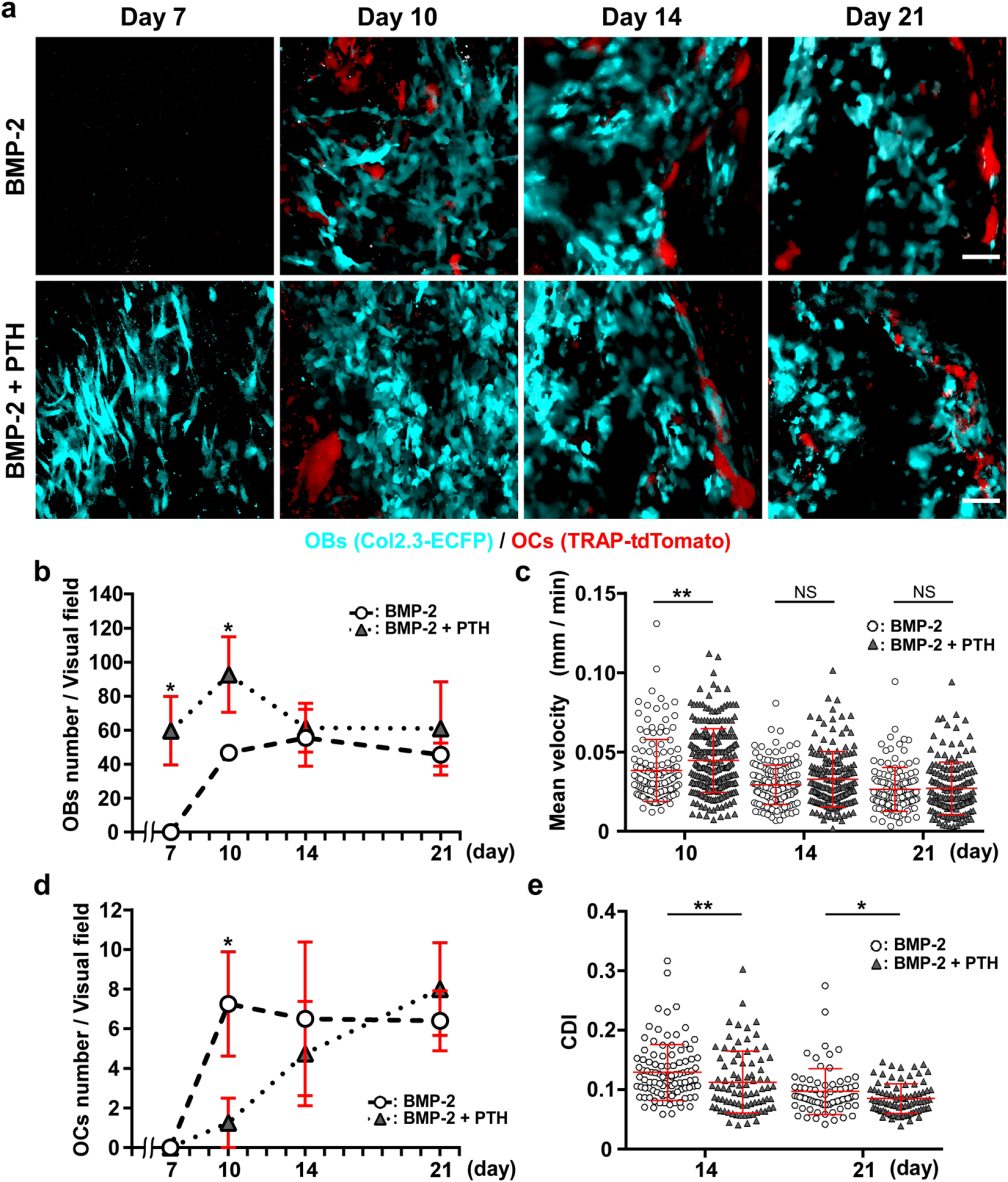
PTH-induced changes in the number and motility of OBs and OCs (**a**) Representative intravital two-photon microscopy images of ectopic bone formation in untreated (upper panels) and PTH-treated (lower panels) *Col2.3*-ECFP/TRAP-tdTomato mice from day 7 to day 21 after CS implantation. Cyan: OBs expressing *Col2.3*-ECFP; red: OCs expressing TRAP-tdTomato. Scale bar, 50 μm. (**b**) Changes in the number of OBs/visual field. n = 4–5/group. (**c**) Mean velocity of OBs/visual field. Data points (without/with PTH treatment, n = 141/253, 167/168, and 129/160 on days 10, 14, and 21, respectively) represent single cells collected from three mice/group. (**d**) Changes in the number of OCs/visual field. n = 4–5/group. (**e**) Changes in CDI of OCs/visual field, with and without PTH treatment. Data points (without/with PTH treatment, n = 100/84 and 72/84 on days 14 and 21, respectively) represent single cells collected from three mice/group. Data are presented as means ± SD. *P < 0.05; **P < 0.01; NS, not significant (Mann-Whitney test).

Finally, we investigated how intermittent PTH administration affects the dynamics of OBs and OCs (Fig. 5a, Supplementary Movie 3). In *Col2.3*-ECFP/TRAP-tdTomato mice that were not treated with PTH, OBs appeared on day 10 and their number peaked on day 14, after which the number declined by day 21. In contrast, after PTH administration, the number of OBs peaked on day 10 and was significantly higher than the numbers measured on days 7 and 10 in the absence of PTH treatment. Furthermore, the OB number in PTH-treated mice did not decrease from day 14 to day 21, whereas the number showed a decreasing trend in untreated mice during the same period (Fig. 5b). Analysis of PTH-induced change in OB motility revealed that the mean velocity of OBs decreased over a period similar to that observed in the absence of PTH treatment, although the velocity at day 10 was significantly higher than that measured in the absence of PTH administration (Fig. 5c). These results showed that PTH administration led to differentiation, with increase in OB number and motility.

In the case of OCs, the cells were fewer in PTH-treated mice than in untreated mice, and the OC number increased gradually over time after PTH administration in contrast to the number measured in the absence of PTH, which remained constant after day 10 (Fig. 5d). Histological evaluation using TRAP staining supported the findings of *in vivo* imaging (Supplementary Fig. S3c). We evaluated the morphological change in OCs (amoeboid movement) using a cell deformation index (CDI) according to a previous report (Supplementary Fig. S5)^26^; low CDI value (static OCs) is reported to correlate with high bone-resorptive activity. Our analysis revealed that CDI values decreased over time both in the presence and absence of PTH administration, although the values were significantly lower in PTH-treated mice than in PTH-untreated mice on days 14 and 21 (Fig. 5e). These results suggested that PTH increases the bone-resorptive activity of OCs in BMP-2-induced ectopic bone.

## Discussion

Advances in imaging technology have enabled intravital two-photon imaging of normal bone as well as bones in several disease models^12–14,26,27^. However, a technique for intravital imaging of ectopic bone formation in soft tissue was not established because of the challenges associated with controlling body motion (breathing and heartbeat) and exposure-related bleeding. In this study, we developed a method for intravital imaging of BMP-2-induced ectopic bone formation, in which a flipped ossified lesion was examined using inverted two-photon microscopy. This technique allowed spatiotemporal visualization of the entire bone formation process, which is initiated by the formation of blood vessels^28^ and is followed by the induction of OBs and OCs, the formation of CFs, and mineral deposition. Similar results were obtained for histological evaluation. Furthermore, we were able to quantify dynamic changes in the motility of OBs and OCs in BMP-2-induced bone, which cannot be evaluated using conventional histomorphometric analyses. Finally, our study revealed that pharmacological intervention using PTH caused earlier differentiation and increased the motility of OBs, whereas it decreased the morphological changes in OCs *in vivo*. Here, we have also proposed three dynamic quantitative parameters for the optimization of ectopic bone formation.

First, we developed methods for quantifying CF orientation, OB morphology, and their correlation. In the early stage of ectopic bone formation, spindle-shaped OBs were recruited and unidirectional CFs were observed, and the OBs and CFs showed orientational correlation. This result agreed with a previous report showing that CF orientation is determined by OB orientation, which, in turn, is regulated by scaffold anisotropy^29^. Furthermore, our RNA-seq results showed that genes associated with ‘organization of the cytoskeleton’ were significantly upregulated between days 10 and 14, which also supported the report that cytoskeletal reorganization depends on the scaffold anisotropy^29^. As the blood vessels that were induced at the early stage were formed towards the center of the implanted CS, and as the endothelium serves as a template on which bone-forming cells build new bone tissue^30^, the blood vessels (endothelial cells) are expected to serve as a scaffold for OBs. The morphology of OBs changed from being spindle-shaped to cuboidal, and the orientation of CFs changed from anisotropic to isotropic over time in accordance with the maturity of the induced bone. These results suggested that the scaffold anisotropy changed to isotropic due to the increase in the volume of the induced bone, which acted as a scaffold for the OBs. Furthermore, at the early stage, PTH administration increased CFOI (isotropic change) and decreased OB eccentricity. These indices can be used as indicators of the maturity of BMP-2-induced bone.

Second, the motility of OBs is expected to reflect the increased recruitment of new OBs caused by the acceleration of the differentiation of mesenchymal stem cells or progenitor cells. Increased OB motility, which precedes the increase in bone volume (Figs. 1c, 2f), can act as an indicator of the subsequent active bone formation. The motility of OBs is expected to be a more sensitive indicator of bone formation than the number of OBs, as the OB number measured may potentially include OBs that are buried in the bone matrix and do not actively contribute to bone formation. The earlier than normal appearance of OBs induced by PTH might be supported by the effects of PTH on Wnt signaling, which accelerates osteogenic differentiation of mesenchymal progenitor cells instead of adipogenic differentiation^19,20,31^, reactivates lining cells^32,33^, and suppresses apoptosis in OBs^19,34^. These findings also suggest that the anabolic effect of PTH is markedly stronger in newly forming bone than the effect observed in bone undergoing normal remodeling.

Third, the dynamic change in OC morphology was quantified using the CDI value, which was found to decrease after PTH administration. Excessive BMP-2 signaling upregulates *DKK1* and *Sost*, which negatively regulates the Wnt pathway^35^. PTH suppresses the negative action of *DKK1* and *Sost* on Wnt receptor and activates Wnt signaling^36,37^. Therefore, the administration of PTH during BMP-2 induced bone formation can lead to synergistic bone formation by enhanced osteoblastic differentiation and suppression of osteoclastogenesis^21^. The PTH-induced decrease in OC number on day 10 can be explained by this mechanism. The increase in the number of OCs following PTH administration might be a secondary effect produced by the increased *RANKL* expression induced by the elevation in OB number^38^.

In this study, PTH treatment decreased CDI values on days 14 and 21. CDI decrease is reported to correlate with the increase in the number of static bone-resorptive OCs^26^. The measurement of CDI can be used as a dynamic indicator of bone remodeling by OCs in BMP-2-induced ectopic bone formation.

*In vivo* quantitative analysis of how and when pharmacological agents that affect ectopic bone formation can facilitate the optimization of this process is lacking. Studies are underway to elucidate the controversial origin of the OBs that contribute to ectopic bone formation using intravital imaging of ectopic bone formation^39^.

In this study, we established a novel method for intravital imaging of BMP-2-induced ectopic bone formation. However, early events of BMP-2-induced bone formation, such as emergence of skeletal stem cells in the capillary vessels or chondrogenesis, were not visualized. Therefore, our future goal of this intravital imaging system for bone formation is the simultaneous visualization of skeletal stem cells and chondrocytes during the early bone formation process. By establishing this model, we hope to elucidate the contribution of skeletal stem cells in the capillary vessels and chondrocytes during bone formation and the effects of pharmacological treatment on skeletal stem cells or chondrocytes during the bone formation^40–42^.

In conclusion, a novel method for intravital imaging of BMP-2-induced ectopic bone formation was established, and the effects of PTH administration on the formation of OBs, OCs, and CFs were quantified *in vivo*. We believe that this imaging method will provide novel insights, which will enhance our understanding regarding the *in vivo* dynamic mechanism of BMP-2-induced bone formation and the methods required to optimize this induction process.

## Materials and Methods

### Mice

Male C57BL/6 J mice were purchased from Clea Japan (Tokyo, Japan). The generation of *Col2.3-*ECFP mice and *Col2.3*-ECFP/TRAP-tdTomato mice (double-fluorescent-reporter mice) has been described previously^13,26^. In *Col2.3*-ECFP mice, ECFP expression is driven by the type I collagen promoter in OBs; in TRAP-tdTomato mice, the expression of the red fluorescent protein tdTomato is driven by the TRAP promoter in OCs. All mice were maintained under specific-pathogen-free conditions and used in accordance with the guidelines of the Institutional Animal Care and Use Committee of Osaka University. All our experimental protocols were approved by the Animal Experimental Committee of Osaka University.

### Surgery for subcutaneous ectopic bone formation

Male *Col2.3*-ECFP and *Col2.3*-ECFP/TRAP-tdTomato mice (12–16 weeks old) were used for the experiments described herein. A degradable CS (Colla Tape Absorbable Collagen; Zimmer Biomet Holdings Inc.) was cut into 4 × 6 mm fragments, and 2.5 μg rhBMP-2 (Osteopharma Inc.) dissolved in phosphate-buffered saline (PBS) was applied into the CS fragments immediately before implantation. The rhBMP-2 dose was determined based on the results of a preliminary study (data not shown). Mice were anaesthetized with isoflurane (Escain; 2.0% vaporized in 100% oxygen), and before surgery, their skin was shaved and disinfected using 70% ethanol. Next, a 10-mm paramedian longitudinal incision was created on the back and subcutaneous tissue and bluntly separated laterally; a silicone sheet (Koken CO., LTD.) was inserted between the fascia and the skin to prevent CS adhesion to surrounding tissue. Finally, the CS was implanted between the silicone sheet and the subcutaneous tissue; the silicone sheet was sutured to the skin, and the wound was closed using stitches (Fig. 1a).

### Intravital two-photon imaging of ectopic bone formation

On days 7, 10, 14, and 21 after CS implantation, mice were anaesthetized as described above, and the skin of the back, including the CS implantation site, was inverted after a U-shaped incision. The implantation sites were exposed by removing the silicon sheet. The inverted skin was fixed on the microscope stage with tape and covered with glass to maintain a wet environment (Supplementary Fig. S1a). An inverted two-photon microscope was used for the following examination. Imaging was conducted at the outer part of the CS where bone formation starts (Fig. 1a). The observed area for the newly formed CFs was the outside of the implanted CS, which was easily distinguishable from the newly formed CFs. During the intravital imaging experiments, the imaging box and the anaesthetized mouse were maintained at a constant warm temperature using heated air. Heart rate was monitored using an electrocardiogram monitor device (Nihon Kohden), and the anesthetic gas concentration was adjusted by using the heart rate as a guide.

### Conditions for two-photon microscopy

The imaging system comprised a Nikon inverted two-photon microscope (A1-MP; Nikon) equipped with a 20× water-immersion objective lens (CFI APO LWD Lambda S 20× WI/0.95; Nikon), and was driven by a laser (Chameleon Vision II Ti:Sapphire; Coherent, Inc.). Multi-fluorescent images were acquired via direct detection of fluorescence using four external non-descanned detectors equipped with dichroics and emission filters: an infrared-cut filter (DM685), three dichroic mirrors (DM458, DM506, and DM561), and four emission filters (417/60 for the SHG image, 480/40 for ECFP, 534/30 for autofluorescence, and 612/69 for tdTomato/rhodamine/alizarin)^13^. The excitation wavelengths were 820 nm for ECFP, rhodamine (for visualization of blood vessels), alizarin (for calcium staining), and SHG in *Col2.3*-ECFP mice; 900 nm for ECFP, tdTomato, and SHG in *Col2.3*-ECFP/TRAP-tdTomato mice. The acquired images were subjected to channel unmixing using NIS Elements integrated software (Nikon) for autofluorescence and crosstalk reduction. Tetramethyl rhodamine (2000 kDa; Invitrogen Co., Ltd.) was intravenously administered before imaging, and alizarin (40 mg/kg; Muto Pure Chemicals CO., LTD.) was intraperitoneally administered the day before imaging.

For intravital time-lapse imaging of the implantation site, image stacks were collected at 3-μm vertical steps at a depth of 50–150 μm, and X–Y resolution of 512 × 512 or 1,024 × 1,024. For imaging CF and OB morphology, the image stacks were collected at 3-μm vertical steps at 50–200-μm depth and 2,048 × 2,048 ×–Y resolution.

Raw imaging data were subjected to channel unmixing for autofluorescence and crosstalk reduction and advanced denoising for noise reduction, and the maximum intensity projection images were corrected for XY drift using the NIS Elements integrated software according to the manufacturer’s standard protocol.

### Intermittent administration of PTH

The murine model of intermittent PTH treatment has been described previously^13,24,25^. Male *Col2.3*-ECFP/TRAP-tdTomato mice (12–16 weeks old) were treated with PTH1-34 (teriparatide; Eli Lilly and Company, 40 μg/kg/day, 5 days/week), which was delivered via subcutaneous injections according to the previous reports^13,24,25^. Injections were initiated 1 week before implantation surgery and were continued until intravital imaging was performed at 7, 10, 14, and 21 days after the surgery.

### Fluorescence activated cell sorting (FACS) analysis

For the analysis and sorting of ECFP^+^ OBs, BMP-2-induced ectopic bone from *Col2.3*-ECFP mice was extracted and minced into 1–2-mm^2^ pieces using scissors. The bone pieces were digested once with 0.25% trypsin for 15 min at 37 °C and then once (day 10) or twice (day 14) with 0.2% collagenase II (Worthington) for 40 min at 37 °C. The cells were collected and resuspended in PBS containing 2% fetal bovine serum and then stained with allophycocyanin-conjugated anti-mouse CD45 antibody (BioLegend), phycoerythrin-conjugated anti-mouse CD45 antibody (eBioscience), and 7-aminoactinomycin D (7AAD) (eBioscience). Hematopoietic cells and dead cells were gated out based on anti-CD45 and 7AAD staining. The stained cells were sorted using a FACSAria II system (BD Biosciences).

### RNA-seq analysis

ECFP^+^ OBs were isolated from ectopic bone using a FACSAria II system, and total RNA was extracted from the collected cells using a miRNeasy kit (Qiagen) according to manufacturer’s instructions. For RNA library preparation, cDNA was generated using the Clontech SMART-Seq v4 Ultra Low Input RNA kit (Takara Clontech, Mountain View, CA, USA). Each cDNA sample was sheared (200–500 bp) on a Covaris S220 (Covaris, Woburn, MA, USA) and prepared using KAPA library preparation kits (Kapa Biosystems, Wilmington, MA, USA). Sequencing was performed on an Illumina HiSeq 2500 platform in the 75-base single-end mode. The Illumina Casava 1.8.2 software was used for base-calling. The raw reads were mapped to the mouse reference genome sequences (mm10) using TopHat ver. 2.0.13 in combination with Bowtie2 ver. 2.2.3 and SAMtools ver. 0.1.19. The number of fragments per kilobase of exon per million mapped fragments (FPKMs) was calculated using Cufflinks ver. 2.2.1^43,44^. Bioinformatics analyses were conducted using Ingenuity Pathway Analysis software (Ingenuity Systems; Qiagen). Raw data obtained in this study have been submitted to the Gene Expression Omnibus (GEO) (accession number: GSE123985).

### OB morphological analysis based on eccentricity

To quantify the changes in OB morphology, we used eccentricity as a measure of cellular shape. Eccentricity is defined by $\sqrt{1-{L}_{minor}^{2}/{L}_{major}^{2}}$, where ${L}_{minor}$ and ${L}_{major}$ are the lengths of the minor and major axes of the cell. The eccentricity of a circle is 0 and that of a straight line is 1. Here, the contours of OBs were manually detected from cropped images using the NIS Elements software, and the eccentricity of each cell was calculated using the computeFeatures function in EBImage (ver. 4.22.1) package.

### CFOI analysis

To quantify changes in CF orientation, we developed a method of image analysis for obtaining the CFOI based on Fourier transformation; 2D Fourier transformation analysis has been widely used to measure CF orientations^45^. We applied the technique to two-photon microscopy images of the SHG channel, and implemented the CFOI method in R (ver. 3.5.1) with the image-processing aspect using the EBImage (ver. 4.22.1) package.

Pre-processing for image analysis was performed as follows. First, small collagen-texture-rich areas (512 × 512 pixels) were cropped from original images (2,048 × 2,048 pixels), and the SHG channels were extracted and converted to grayscale. Next, the images were windowed using a Gaussian profile^46^ to avoid ‘edge effects’ in subsequent Fourier analysis, and the power spectrum was obtained using 2D Fourier transformation and converted to cylindrical coordinates.

CFOI, which indicates the degree of CF orientation, was calculated from low-pass-filtered cylindrical Fourier spectra. Before calculating the CFOI, a low-pass filter was used to remove high-frequency components, such as undulation on the CFs or noise. In this case, the high cut-off frequency was set at 10 pixels (~3.2 μm). CFOI is the ratio of the sum of magnitude for the major orientation to that for the minor orientation. Here, we defined major orientation as the direction that maximized the sum of spectral magnitude, including plus and minus 45°; minor orientation was defined as the diagonal direction of the major orientation. CFOI ranged from 0 to 1; values close to 1 indicated random distribution of CFs in the field of view, whereas low values indicated an increased population of parallel-oriented CFs.

### Orientational correlation between CFs and OBs

CF orientation relative to OB orientation was quantified using a cross-correlation method. CF orientation was characterized as the sum of the magnitude for each angle (as described above). This sequence of the sums of magnitude is represented as $f(\theta )$, $0\le {\rm{\theta }} < 360$. Conversely, the characteristics of OB orientation were determined by calculating the average of the cell radius for each angle, $g(\theta )$, $0\le {\rm{\theta }} < 360$. Cross-correlation analysis between $f(\theta )$ (sum of fiber magnitude for each angle) and $g(\theta )$ (average of cell radius for each angle) revealed directional relationships, and we defined OCI as the correlation value at 90° rotation, ${\int }^{}f(\theta )g(\theta +\tau )d\theta$, where $\tau =90$. High OCI indicates that CF and OB orientations are both anisotropic and that they are orthogonal.

### Analysis of OB motility

OB motility was analyzed using the Imaris software (Bitplane). In the intravital time-lapse imaging of ectopic bone formation in *Col2.3*-ECFP and *Col2.3*-ECFP/TRAP-tdTomato mice, the OBs were tracked semi-automatically at 10-min intervals for 3 h. A dynamic parameter (mean velocity) was directly obtained from the software.

### CDI analysis for quantifying osteoclastic activity

OC morphological changes were quantified based on the CDI using the image analysis software CL-Quant 2.30 (Nikon) to track the changes in OC morphology^26^. Cell shapes were semi-automatically recognized by the software, and the CDI was calculated as the ratio of the cell areas changed over 10 min. CDI decrease is reported to correlate with the high bone-resorptive activity of OCs.

### Tissue preparation and staining

The tissue samples of ectopic bone were fixed in neutral buffered formalin, decalcified, infiltrated, embedded in paraffin wax, and sectioned at a thickness of 5 μm^21,47^. The sectioned samples were deparaffinized, and then stained with hematoxylin and eosin (H & E) using standard procedures. TRAP staining was performed using a TRAP staining kit (Cosmo Bio), according to the manufacturer’s protocol^48^.

### Micro-CT

Micro-CT was used to quantify the volume of induced bone at 7, 10, 14, and 21 days after surgery. Samples were scanned with R_mCT (Rigaku Mechatronics); scanning was performed using a source voltage of 90 kV and a source current of 200 μA. Visualization and data reconstruction were performed using the TRI/3D-BON software (RATOC System Engineering).

### Statistical analysis

Statistical analysis, unless otherwise indicated, was performed using GraphPad Prism (GraphPad Software, Inc.). All results are presented as means ± standard deviation (SD) and were analyzed using Mann–Whitney test for between-group comparisons; the Kruskal-Wallis test with Dunnett’s multiple-comparisons *post hoc* test was used for comparisons among ≥ 3 groups. All data shown are representative of those from at least three independent experiments. P < 0.05 was considered significant.

## Supplementary information

Supplementary Information.

Supplementary Information2.

Supplementary Information3.

Supplementary Information4.

## Data Availability

Data supporting the findings of this study are available within the paper and its Supplementary Information. RNA-seq data generated in this study are available through the GEO (accession number: GSE123985).
