# Supplementary material for: In vivo dynamic analysis of BMP-2-induced ectopic bone formation
Source: Sci Rep. 2020 Mar 16;10:4751. doi: 10.1038/s41598-020-61825-2 (PMC7076033; doi:10.1038/s41598-020-61825-2)
Supplement: Supplementary file 4 — Supplementary Information4. [file 41598_2020_61825_MOESM4_ESM.pdf]

In vivo dynamic analysis of BMP-2-induced ectopic bone formation

Kunihiko Hashimoto[1,2], Takashi Kaito[2], Masayuki Furuya[1,2,3], Shigeto Seno[4], Daisuke Okuzaki[5], Junichi Kikuta[1], Hiroyuki Tsukazaki[1,2], Hideo Matsuda[4], Hideki Yoshikawa[2], and Masaru Ishii[1]

[1]Department of Immunology and Cell Biology, Graduate School of Medicine & Frontier Biosciences, Osaka University, Osaka 565-0871, Japan

[2]Department of Orthopaedic Surgery, Graduate School of Medicine, Osaka University, Osaka 565-0871, Japan

[3]Department of Orthopaedic Surgery, Japan Organization of Occupational Health and Safety Osaka Rosai Hospital, Osaka 591-8025, Japan

[4]Department of Bioinformatic Engineering, Graduate School of Information Science & Technology, Osaka University, Osaka 565-0871, Japan

[5]Genome Information Research Center, Research Institute for Microbial Diseases, Osaka University, Osaka 565-0871, Japan

Content of Supplementary information

Supplementary figure S1. The method for ectopic bone imaging

Supplementary figure S2. Tracking, FACS, and RNA sequence analysis of OBs

Supplementary figure S3. Micro-CT images and histological sections of ectopic bone

Supplementary figure S4. CF formation and OB morphology without or with PTH administration

Supplementary figure S5. CDI analysis

Supplementary Movie 1. Intravital two-photon imaging of BMP-induced bone in Col2.3-ECFP mice with visualized blood vessels

Supplementary Movie 2. Intravital two-photon imaging of BMP-induced ectopic bone formation process in Col2.3-ECFP mice with visualized blood vessels

Supplementary Movie 3. Intravital two-photon imaging of OBs and OCs during the BMP-induced ectopic bone formation process in *Col2.3*-ECFP/TRAP-tdTomato mice treated with or without PTH

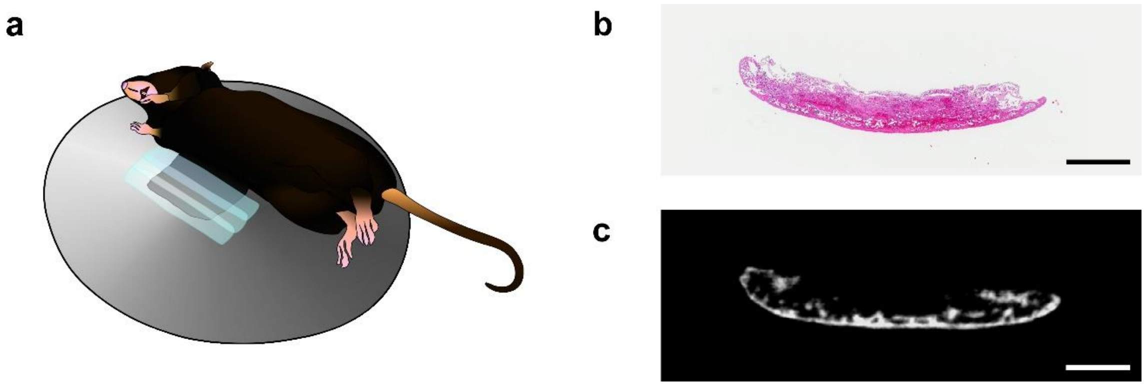

Supplementary figure S1. The method for ectopic bone imaging

(**a**) Schematic presentation of the method for fixing the flipped skin on the imaging table and observing the intravital ectopic bone formation under the microscope stage. The image was created by Canvas software (version X 16, https://www.canvasgfx.com/). (**b**) A representative H & E stained section of the newly formed bone on day 14. Scale bars, 1 mm. (**c**) A micro-CT image of the ectopic bone on day 14. Scale bars, 1 mm. Images of H & E stained section and micro CT is shown in a manner such that the top is the side where silicon sheet was inserted.

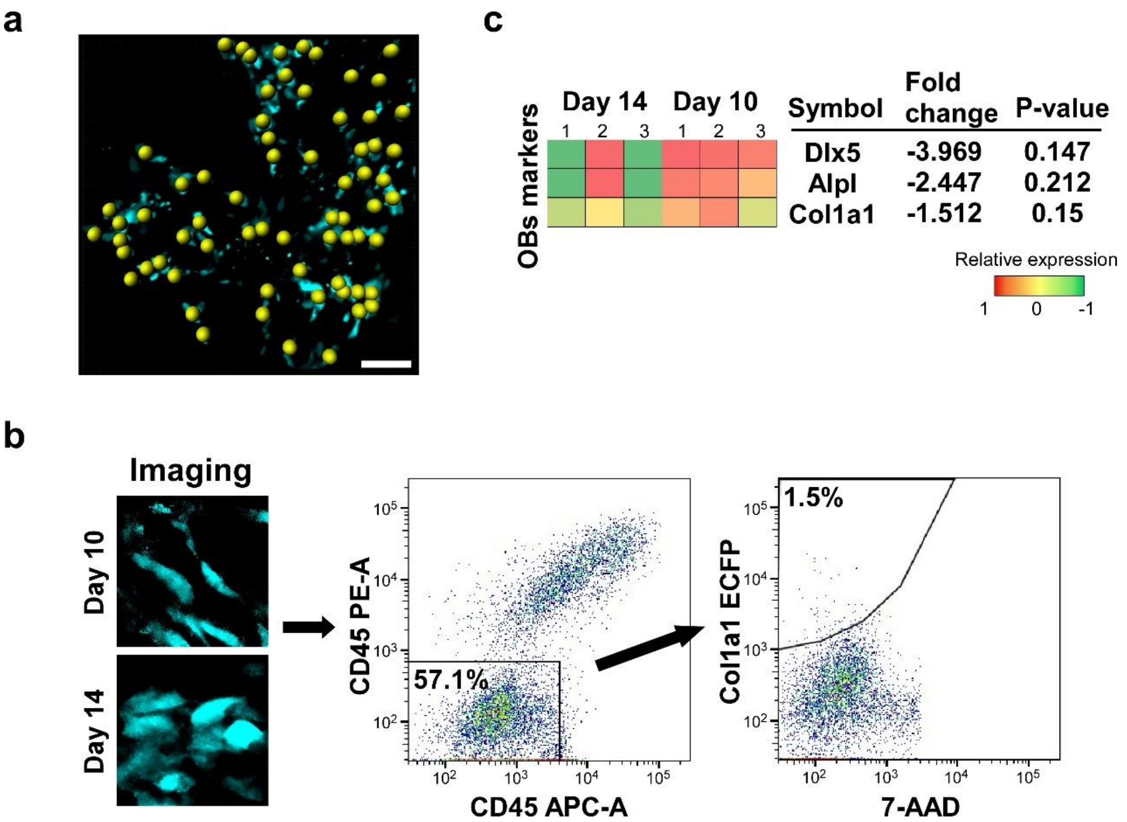

Supplementary figure S2. Tracking, FACS, and RNA sequence analysis of OBs

(**a**) The motility of OBs was tracked at 10-min intervals for 3 h. Scale bar, 50 µm. Created the images using Imaris software (version 9.0, https://imaris.oxinst.com/) (**b**) Representative flow cytometry dot plot analysis showing the percentage of ECFP^{+} cells in ectopic bone from *Col2.3*-ECFP mice on days 10 and 14. APC, allophycocyanin; PE, phycoerythrin. (**c**) Heat map showing expression levels for genes related to osteoblastic differentiation on day 10 and day 14 in ectopic bone. n = 3/group. Data are presented as means ± SD. (Mann-Whitney test). The image was created Canvas software (version X 16, https://www.canvasgfx.com/).

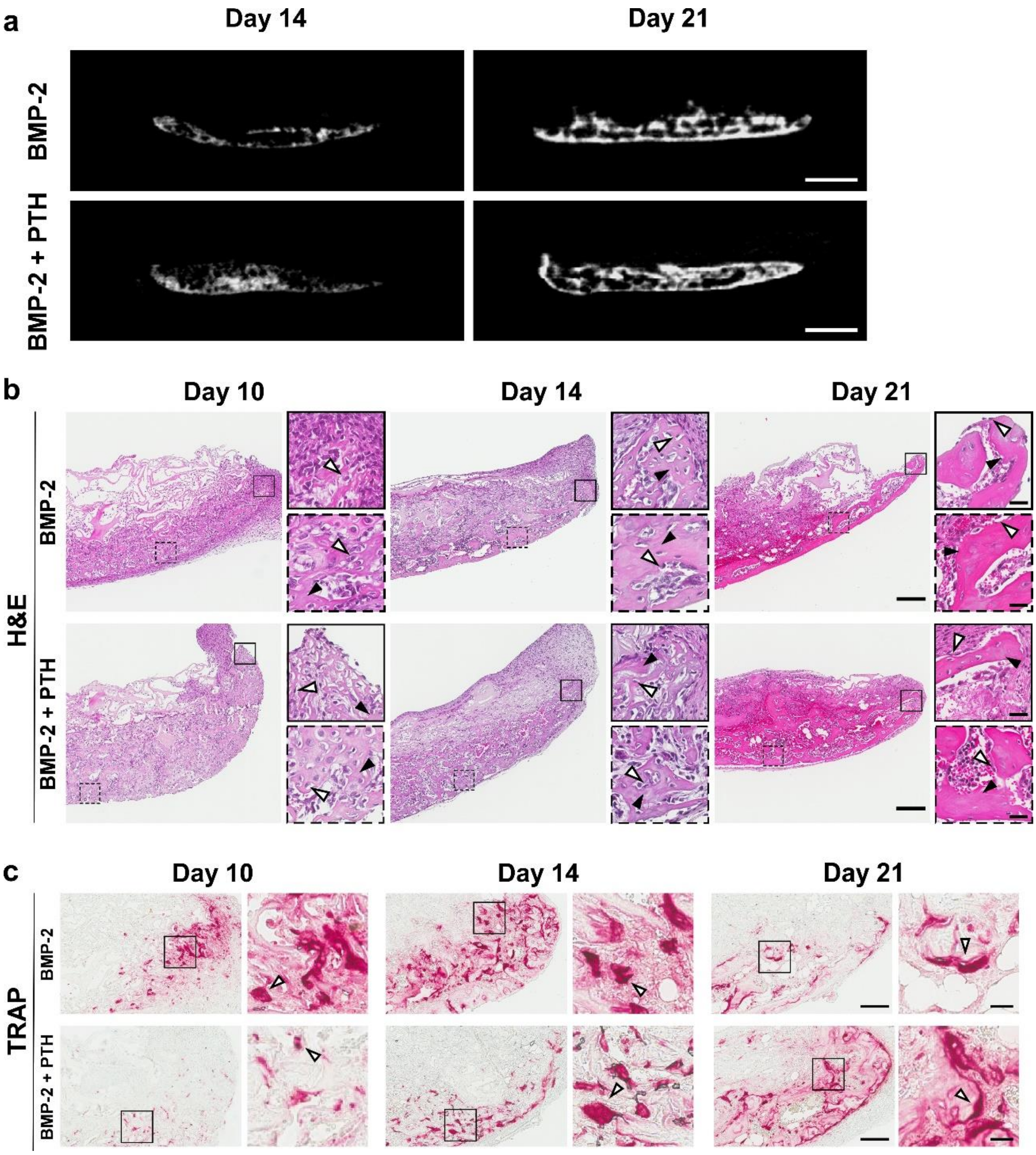

Supplementary figure S3. Micro-CT images and histological sections of ectopic bone

(**a**) Representative micro-CT images of ectopic bone on days 14 and 21. Scale bars, 1 mm. (**b**, **c**) Representative histological sections of ectopic bone in *Col2.3*-ECFP/TRAP-tdTomato mice without and with PTH treatment on day 10 to 21.

Two magnified images of the region under observation using two-photon microscopy (region outlined) and the region where bone formation is in progress (region delineated by dotted lines) are shown. (**b**) Hematoxylin and eosin (H & E) staining of ectopic bone. OBs (open arrowheads) and bone formation (filled arrowheads). Scale bar, 200 µm and 25 µm (magnified images). (**c**) Representative TRAP-stained histological sections of ectopic bone. OCs, open arrowheads. Scale bars, 100 µm and 20 µm (magnified images).

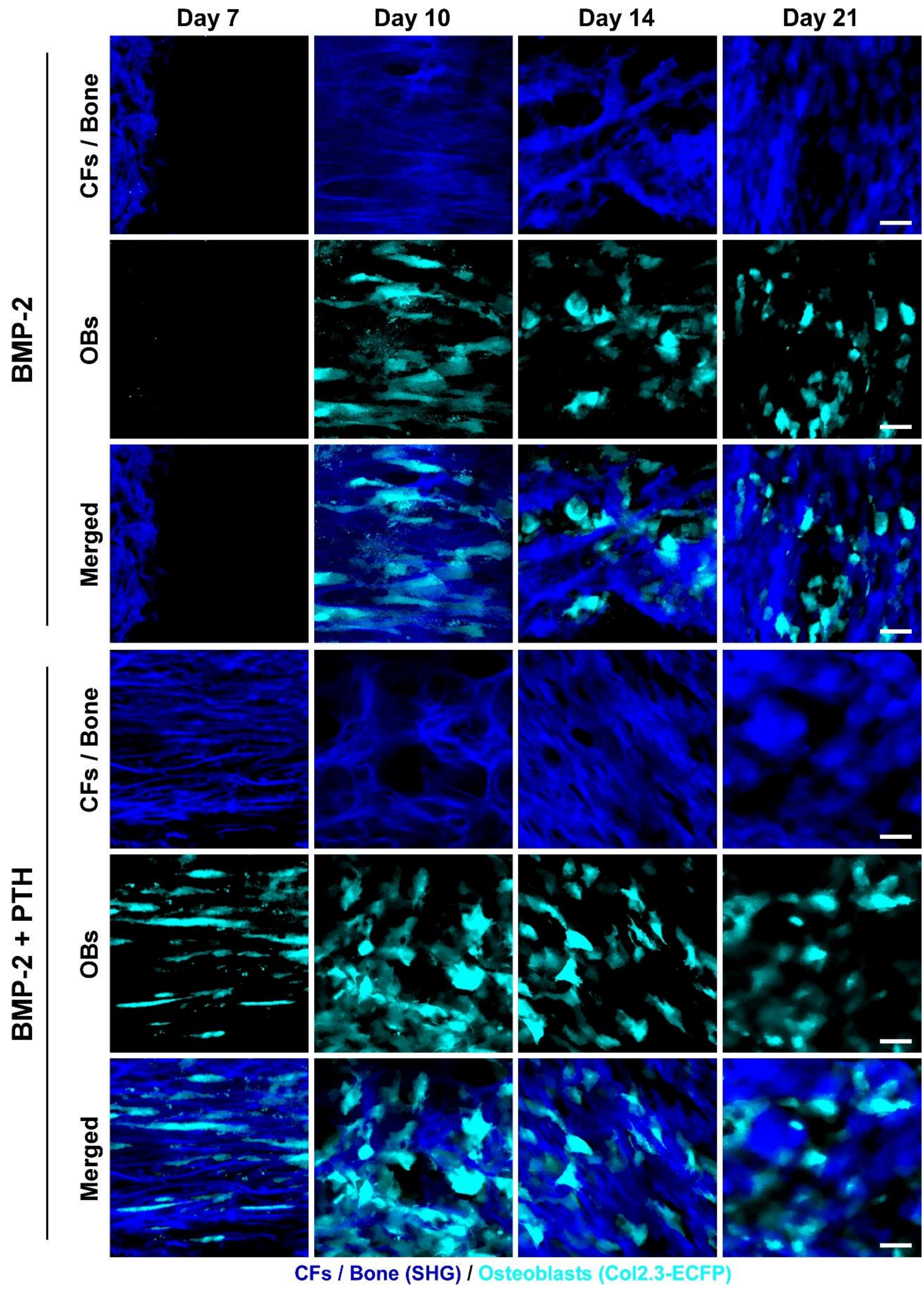

Supplementary figure S4. CF formation and OB morphology without or with PTH administration

Temporal change of CF formation and OB morphology in *Col2.3*-ECFP/TRAP-tdTomato mice without (upper 3 panels) or with (lower 3 panels) PTH treatment from day 7 to 21 after CS implantation. Blue, CFs / Bone (SHG); cyan, OBs expressing *Col2.3*-ECFP. Scale bar, 25 µm.

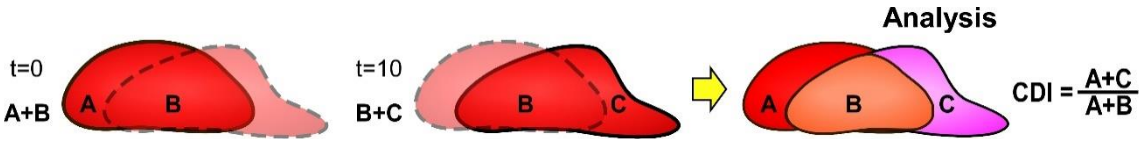

Supplementary figure S5. CDI analysis

Formula of CDI analysis for OCs. Cell shapes were semi-automatically recognized by the image analysis software, and three distinct areas were defined as follows: the area that was occupied in only the initial time frame (t = 0) (A), the area that was occupied in only the final time frame (t = 10) (C), and the overlapping area in the initial and final frames (B). The CDI was calculated as (A + C) / (A + B) and represents the ratio of the cell areas changed during the 10-min interval. Low CDI value correlates with the low motility and high bone resorptive activity of OCs. The images were created by Canvas software (version X 16, https://www.canvasgfx.com/).

Supplementary Movie 1. Intravital two-photon imaging of BMP-induced bone in Col2.3-ECFP mice with visualized blood vessels

Mice were treated with tetramethyl rhodamine via intravenous injection before imaging. Sequential images in the same visual field were acquired under control conditions. Cyan: OBs expressing Col2.3-ECFP; red: blood vessels stained with rhodamine; blue: CFs/bone (SHG). Scale bar, 100 μm. Playback speed, 3600×.

Supplementary Movie 2. Intravital two-photon imaging of BMP-induced ectopic bone formation process in Col2.3-ECFP mice with visualized blood vessels

Mice were treated with tetramethyl rhodamine via intravenous injection before imaging. Sequential images in the same visual field were acquired under control conditions. Cyan: OBs expressing Col2.3-ECFP; red: blood vessels stained with rhodamine; blue: CFs/bone (SHG). Scale bar, 100 μm. Playback speed, 1800×.

Supplementary Movie 3. Intravital two-photon imaging of OBs and OCs during the BMP-induced ectopic bone formation process in *Col2.3-*ECFP/TRAP-tdTomato mice treated with or without PTH

Mice were treated with PTH (40 μg/kg/day, 5 days/week) via subcutaneous injections. Sequential images in the same visual field were acquired. Cyan: OBs expressing Col2.3-ECFP; red: OCs expressing TRAP-tdTomato; blue: CFs/bone (SHG). Scale bar, 100 μm. Playback speed, 3600×.
